# Spectroscopic Ellipsometry of Conducting Anisotropic Pedot thin Films

**DOI:** 10.1002/marc.202500965

**Published:** 2026-03-30

**Authors:** Francesco Bisio, Katia Sparnacci, Angelo Angelini, Martina Martusciello, Daniela Di Fonzo, Davide Comoretto, Maddalena Patrini

**Affiliations:** ^1^ Dipartimento Di Scienze e Innovazione Tecnologica Università degli Studi del Piemonte Orientale Alessandria Italy; ^2^ Advanced Materials Metrology and Life Science Division INRiM Institute (Istituto Nazionale Di Ricerca Metrologica) Torino Italy; ^3^ Dipartimento di Chimica e Chimica Industriale Università degli Studi di Genova Genova Italy; ^4^ Dipartimento di Fisica Alessandro Volta Università degli Studi di Pavia Pavia Italy

**Keywords:** anisotropy, conducting polymers, ellipsometry, optical functions, PEDOT, thin films

## Abstract

We report a comprehensive spectroscopic investigation of poly[3,4‐ethylenedioxythiophene] (PEDOT) thin films, including both those synthesized from solution in the doped state and from commercial PEDOT:PSS (poly(styrene sulfonate)) formulations. Variable‐angle spectroscopic ellipsometry is combined with transmittance and reflectance measurements spanning the UV to mid‐infrared spectral range. Data from the different techniques are consistently analyzed within a unified framework based on the complex dielectric function of each film. Uniaxial optical modeling assuming an edge‐on molecular orientation reveals a markedly anisotropic response, with the ordinary (in‐plane) component exhibiting significantly higher conductivity than the out‐of‐plane dielectric component. Notably, the synthesized PEDOT films show enhanced in‐plane optical conductivity, in agreement with sheet resistance measurements. The molecular structure and anisotropy of PEDOT are further corroborated by grazing‐incidence wide‐angle X‐ray scattering (GIWAXS), which reveals an intra‐lamellar structure characterized by π–π stacking of PEDOT chains at a distance of approximately 3.9 Å and an interlamellar periodicity of about 15.7 Å. Overall, these results represent a significant step toward a deeper understanding of the electronic and optical properties of this distinctive class of macromolecules and their potential use in photonics and optical metamaterials.

## Graphical abstract

We measure the optical response of poly[3,4‐ethylenedioxythiophene] thin films through the combination of polarized photon spectroscopies (variable‐angle ellipsometry, UV–mid‐IR reflectance and transmittance) and wide‐angle X‐ray scattering, revealing anisotropic optical response and conductivity and enabling structural modelling of chain and π‐system orientations within a lamellar microstructure.

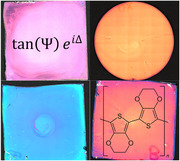

## 1 Introduction

Over the past years, organic nanophotonics—i.e., the subfield of photonics in which light is manipulated and processed using organic semiconductors and devices—has revealed unexpected properties that are unattainable with conventional inorganic materials, such as flexibility, shape adaptability, and stretchability [1, 2, 3, 4, 5, 6, 7]. Optical waveguides [8, 9, 10], optical cavities [11, 12, 13, 14, 15, 16, 17, 18], filters [19, 20], lasers [21, 22, 23, 24, 25, 26, 27], thermal shields [28, 29] photonic crystals [17, 30, 31]. and metamaterials [32, 33, 34] validate the interest fostered by organic photonics. Focusing on lithography‐processed metamaterials, selecting suitable organic materials remains a significant challenge. To date, elastomeric systems have been predominantly employed to impart flexibility or tunability to devices [35, 36], whereas only a limited number of studies have explored the use of functional active polymers as meta‐atoms for constructing metamaterial architectures [7, 37, 38, 39, 40, 41].

From a materials perspective, the development of metamaterials primarily relies on two approaches: the use of materials with very high refractive index (typically exceeding 2) or materials exhibiting metallic character. The former are particularly difficult to identify among macromolecular commodity systems, due to stringent constraints on synthesis and processing. As a result, current research in the field has largely shifted toward hybrid nanocomposites, conjugated systems, and inorganic polymers, such as inverse‐vulcanized ones [42, 43]. As an alternative to metallic structures, conducting polymers represent an up‐and‐coming option. Among them, PEDOT and its derivatives and formulations are especially attractive, as their synthesis is not energy‐intensive, can be carried out at low temperatures, and yields materials that are readily processable from solutions using simple techniques. PEDOT‐based materials have therefore been widely introduced as transparent electrodes in organic electronics [41, 44]. Beyond its use as a transparent electrode, PEDOT has found widespread application in bioelectronics [45], biosensing [46], and thermoelectric devices [47].

For nanophotonic applications, the use of any material—including PEDOT—requires a deep and detailed understanding of its optical response (i.e., the complex refractive index $\tilde{n}=n+ik$, or complex dielectric constant $\left(\tilde{\varepsilon }=\varepsilon_{1}+i\varepsilon_{2}\right)$. This, in turn, is governed by film morphology, which depends on the adopted synthetic strategy, processing conditions, and the counter‐ions used [44, 45, 48, 49]. Notably, the morphology may induce anisotropy in the electrical conductivity [50], thereby indicating a corresponding anisotropy in the electronic structure, which should also be reflected in the optical response.

Within this field, this paper reports on the preparation of self‐doped PEDOT thin films, as obtained via oxidative chemical polymerization in the liquid phase. These samples were obtained using Vanadium pentoxide (V_2_O_5_) as an oxidant and following a procedure adapted from the work of Chen et al. [51] and described in the Experimental Section. As the properties of PEDOT prepared by this unusual synthetic procedure have been rarely investigated, we performed a detailed ellipsometric analysis, combined with broadband optical characterization and extended electrical analysis, which allowed us to unambiguously determine the uniaxial anisotropy of the refractive index, in full agreement with preliminary GIWAXS investigations. The results obtained for PEDOT are then compared with corresponding optical data for commercial PEDOT:PSS formulations, indicating that anisotropy in both the optical and electrical responses is a common characteristic of PEDOT thin films. The results reported here provide the foundation for nanostructuring these films to achieve metamaterial properties.

## 2 Results and Discussion

Three different PEDOT thin film series were prepared on different substrates, namely high‐grade quartz glass, amorphous glass, and intrinsic Silicon slides. The first two sample series were obtained by spin coating starting from commercial PEDOT:PSS formulations (hereafter named SA and OS, after the Sigma‐Aldrich and Ossila Ltd providers, respectively). The third sample series, named UPO, was obtained through a liquid‐phase oxidative chemical polymerisation process using vanadium pentoxide (V_2_O_5_) as the oxidant (see Experimental section/Methods section for details).

**FIGURE 1:**
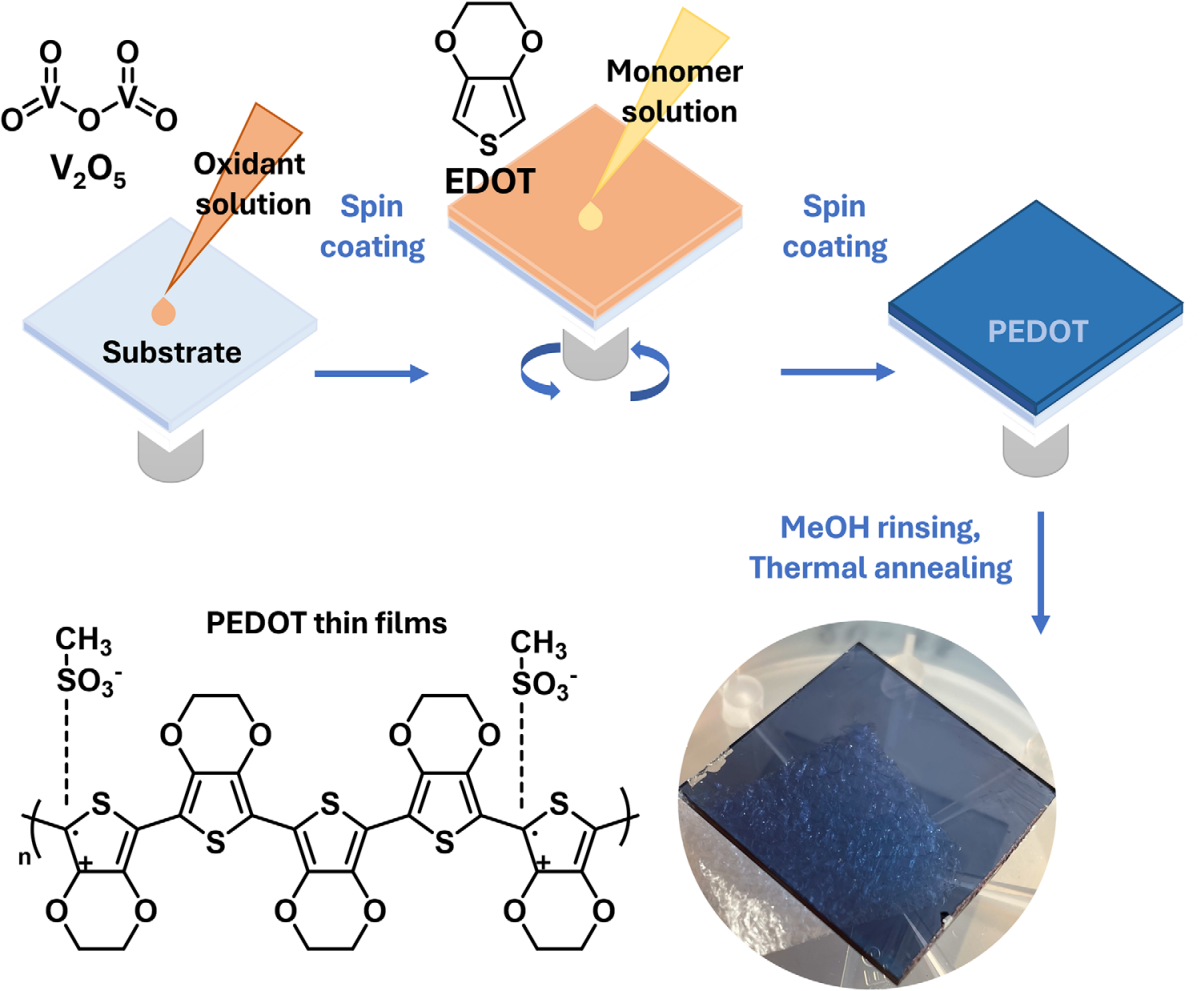
Scheme of the synthesis process used in the preparation of PEDOT UPO samples.

The synthetic process is illustrated in Figure 1. First, an oxidant solution is deposited by spin‐coating onto the treated substrate. Then, without stopping the rotation, a monomer solution is deposited onto the oxidant‐covered substrate, and spin coating is continued for a further 4 s. Under these conditions, polymerisation occurs almost instantaneously, as evidenced by the sample's rapid colour change from yellow (the colour of the oxidising solution) to dark blue (the colour of PEDOT film, Figure 1) immediately after the monomer is added. The oxidant solution consists of V_2_O_5_ dissolved in methanesulfonic acid (MSA), to which a weak base (pyridine) is added to prevent unwanted acidic side reactions [52]. Two solutions are prepared with different concentrations of V_2_O_5_ in MSA: 0.2 and 0.3 m. Unfortunately, V_2_O_5_ dissolves very slowly in MSA, taking approximately three months to reach a concentration of 0.3 m. A third, more concentrated, solution (0.4 m) was obtained by dissolving V_2_O_5_ in a 3:1 (by volume) mixture of MSA and concentrated sulphuric acid [53]. The monomer solution is made up of 30% v/v EDOT dissolved in acetonitrile. Starting from the three oxidant solutions and varying the process times, four sample sets were prepared, with thickness values ranging from 100 to 380 nm, as reported in Table 1.

**TABLE 1:** Synthetic details for the preparation of UPO PEDOT thin films.

Sample Set	Spin‐coating velocity [rpm]	Oxidant solution concentration [M]	Oxidant solution casting time [s]	Film thickness by optical profilometry [nm]
S1	3000	0,4^a)^	6	380
S2	3000	0,2	6	110
S3	3000	0,3	6	200
S4	3000	0,2	7	100

^a)^ V_2_O_5_ dissolved in a mixture 3:1v/v of MSA and concentrated sulphuric acid.

Film thickness values were determined by step‐height analysis using an optical profilometer equipped with a confocal microscope (Figure S1) and compared with those derived by spectroscopic ellipsometry (Table 2, vide infra Table S1 and corresponding comments). Surface roughness was determined both by confocal optical profilometry (Figure S2) and Atomic Force Microscopy (AFM) analysis (Figure S3). The first one indicates a rms roughness value of about 4 nm, while AFM provides 1.7 nm. The thickness of the resulting films is primarily related to the concentration of the oxidant solution used, with the higher concentration resulting in thicker films when the spinning velocity is kept fixed.

**TABLE 2:** Thin film growth, structural, and optical parameters (Γ, ω*_p_*, Σ^D^ / Σ^tot^), for a typical sample of three PEDOT series, whose dielectric functions are reported in Figure 3, both for in‐plane and out‐of‐plane components. Comparison of equivalent values from literature references is also reported.

			Static optical conductivity σ (ω = 0)				In‐plane response			Out‐of‐plane response		
Sample series	Spin coating speed	Film thickness by SE	in‐plane	out‐of‐plane	$\sigma^{in}/\sigma^{out}$	4PP sheet conductivity	$\omega_{p}^{2}$	Γ	Σ^D^ / Σ^tot^	$\omega_{p}^{2}$	Γ	Σ^D^ / Σ^tot^
	[rps]	[nm]	[10^3^ S/m]	[10^3^ S/m]		[10^3^ S/m]	(eV^2^)	(eV)		(eV^2^)	(eV)	
SA	60	142	1.47	0.141	10,5	0.3	0,73	1.06	0.46	0.33	4.97	0.35
OS	60	162	2.26	0.047	48	0.2	0,85	0.80	0.69	0.14	6.22	0.03
UPO	50	108	14.8	1.04	14	33.7	5,52	0.80	0.93	0.36	0.75	0.41
Ref. [60]*		180	700	32	22	670						
Ref. [64]			10	1								

* See also Reference values in Table S1

Variable‐angle spectroscopic ellipsometry (SE) joined to Transmittance/Reflectance dispersive spectroscopy at near‐normal incidence was used to characterize the dielectric response in the UV–vis‐NIR spectral range. Polarized Fourier Transform Infrared spectroscopy in the medium infrared range (up to 0.1 eV) at variable angles of incidence provided further information about birefringence in the optical conduction response. In the static limit, this last allows the comparison to electrical sheet conductivity values obtained by four‐Point Probe (4PP) characterization. Finally, Grazing Incidence Wide‐Angle X‐ray Scattering (GIWAXS) pattern on the UPO series material was used to correlate the optical birefringent model and the molecular backbone orientation within a lamellar ordering.

### 2.1 Optical Spectroscopies: Dielectric and Conduction Response

#### 2.1.1 UV–Vis‐NIR Spectroscopic Ellipsometry STUDY

Spectroscopic ellipsometry (SE) is the method of choice to determine the dielectric function dispersion of a material. Upon reflection at a plane surface, linearly polarised radiation generally becomes elliptically polarised. The measured ellipsometric parameters, tanΨ and cosΔ are the relative amplitude ratio and relative phase shift, respectively, of the complex Fresnel reflection coefficients *r_p_* and *r_s_*, related to the *p*‐ and *s*‐polarised electric field components in the plane of incidence [54]. SE then provides polarisation degree and phase as well as the amplitude information of the optical response within a single experiment; moreover, by collecting spectra at different angles of incidence, the optical modelling, simulation, and quantitative interpretation are improved [55, 56].

SE is used to investigate the relative role of the PEDOT dielectric response (from bound charges), with respect to the conductive one (by quasi‐free charge carriers), and to assess the in‐plane and out‐of‐plane thin film anisotropy. To this end, the semiclassical dielectric response modelling from Lorentz (Equation 1) and Drude (Equation 2) oscillators has been adopted [57]:

(1) $$\tilde{\varepsilon }\left(\omega \right)=\varepsilon_{\infty }+\sum_{j}\frac{A_{j}}{\left(\omega_{0,j}^{2}-\omega^{2}\right)-i\Gamma_{j}\omega }$$

(2) $$\tilde{\varepsilon }\left(\omega \right)=\varepsilon_{\infty }-\frac{{\omega_{p}}^{2}}{\omega^{2}+i\Gamma \omega }$$

where $\varepsilon_{\infty }$ is the residual dielectric function, *A_j_*, ω*_j_* and Γ*_j_* are the oscillator strength, the energy resonance and broadening, respectively, of any interband transition or vibrational mode; ω*_p_* and Γ are the plasma frequency and energy broadening of the collective charge carrier plasma, respectively. The overall Drude‐Lorentz dielectric function model provides accurate insights into the dielectric properties $\varepsilon_{1}\left(\omega \right),\;\varepsilon_{2}\left(\omega \right)$ of the material, including σ(ω), DC conductivity, and anisotropic mobility. In fact, the optical conductivity is defined as:

(3) $$\sigma \left(\omega \right)=\omega \varepsilon_{2}\left(\omega \right)=\frac{\Gamma \omega_{p}^{2}}{\omega^{2}+\Gamma^{2}}\underset{\omega \to 0}{=}\varepsilon_{0}\frac{\omega_{p}^{2}}{\Gamma }=\sigma_{DC}$$

which for ω=0 coincides with the static electrical conductivity value. The in‐plane static conductivity component should be of interest compared to the sheet‐conductivity value derived from the 4PP measurements. These have been derived from the experimental 4PP sheet resistivity *ρ_s_* and the film thickness *t* derived from ellipsometry, through the usually adopted expression σ = 1/(*ρ_s_ t)* [58].

In the best‐fit analysis to SE spectra, we adopted a three‐phase optical system model: ambient (air), thin film, and substrate. Reference samples of quartz glass, amorphous glass, and silicon substrates were characterized by SE before thin film investigation, and their derived dielectric functions are then adopted in the above model. We underline that no need for a roughness overlayer correction is noticed for the samples investigated, according to the good film surface flatness (see Figures S2 and S3, as well as the comment in Section 2). SE spectra are then complex functions of the angles of incidence, the film dielectric function $\tilde{\varepsilon }=\varepsilon_{1}+i\;\varepsilon_{2}=\varepsilon_{0}\;{\tilde{\varepsilon }}_{r}=\varepsilon_{0}{\tilde{n}}^{2}$, and its thickness (*d*).

The material complex dielectric function has been modelled with a set of physical oscillators, with variable lineshape depending on the optical resonance type, that guarantee Kramers‐Kronig consistency. Free‐variable parameters of the fit are then the amplitude, the resonance energy, and the linewidth for the Lorentzian oscillators, the plasma frequency and broadening, beyond film thickness. Then, the simulated SE, R, and T spectra were best fit to the corresponding experimental ones through minimization of the reduced root *χ* ^2^ figure of merit.

As a starting point, we describe the UV–vis‐NIR PEDOT:PSS film response, comparing the results with those previously reported where the effect of the doping on the electronic structure has been mentioned [59, 60]. As it often appears in conjugated polymers, optical anisotropy has to be considered due to the intrinsic electronic anisotropy of π electrons delocalized over polymer backbones. However, a few reliable attempts to investigate the anisotropy of the optical response of conjugated polymer thin films and polymer nanostructures have been reported [55, 61, 62, 63, 64, 65, 66]. To this end, we first described the thin film as a uniaxial system in the plane of incidence frame of reference, with parametrized in‐plane (ordinary) and out‐of‐plane (extraordinary) optical functions.

**FIGURE 2:**
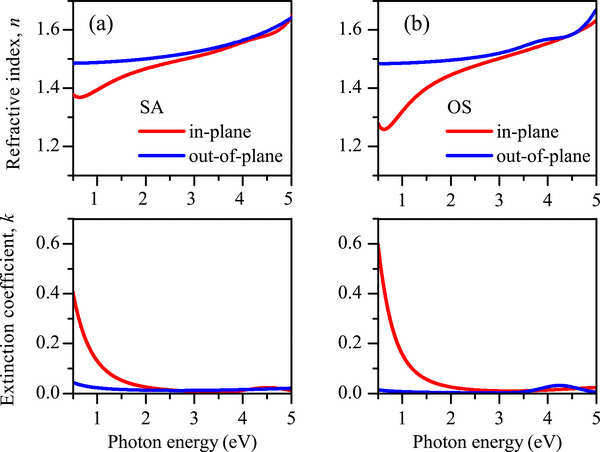
In‐plane and out‐of‐plane components of the complex refractive index—real (*n*, upper panels) and imaginary (*k*, lower panels) part—for SA (a, left) and OS (b, right) thin films.

In Figure 2 we report the complex refractive index dispersion $\left(\tilde{n}=n+ik\right)$ assessed by the best‐fit to the experimental spectra for the two commercial SA and OS PEDOT:PSS thin film series. In both cases, in‐plane refractive index dispersion is stronger than the out‐of‐plane one toward the low energy region, indicating a metallic‐like behaviour. A slight refractive index anisotropy *Δn* of about 0.027 and 0.038 for SA and OS, respectively, is observed at the central visible frequencies (@2.254 eV = 550 nm). The refractive index behaviour reflects the dispersion of the corresponding extinction coefficient, and the conductive response is clearly confirmed in the lower energies in‐plane data sets, with a stronger response for low energies. In addition, the investigated films are spin‐coated from solution, and the evidence from these data is that the incorporated polymer chain backbone (x axis in Figure 5 scheme) and π stacking direction (y axis in the Figure 5 scheme)—where electronic conduction preferentially takes place—align in the substrate plane.

Similar considerations can be drawn for PEDOT of UPO series: its refractive index dispersion is reported for comparison in Figure S4. Note the conductive contribution is stronger (see the n, k scale change); this behavior is deeply discussed in the next subSection.

The SE spectra best‐fit also enables the determination of film thickness, which value was compared with that obtained by optical profilometry (Table S1). We observed excellent agreement between the two techniques, with discrepancies in the values obtained of less than 15%. Nevertheless, we consider the SE results to be more reliable for determining local thickness values. Optical profilometry, in fact, provides an average value over the whole film surface of the film, which is affected by minor thickness inhomogeneities (slight colour variations on the film surface, see Figure S1). This is due to the complicated film formation process, in which polymerisation occurs very quickly during the spin coating process.

On the other hand, thickness and optical functions obtained by spectroscopic ellipsometry are measured over a spot of 200–300 microns in diameter (depending on the incidence angle). Then, SE is probing a more uniform film surface area. For this reason, we consider more reliable the SE results for the local thickness values.

#### 2.1.2 Full Spectroscopic Analysis

**FIGURE 3:**
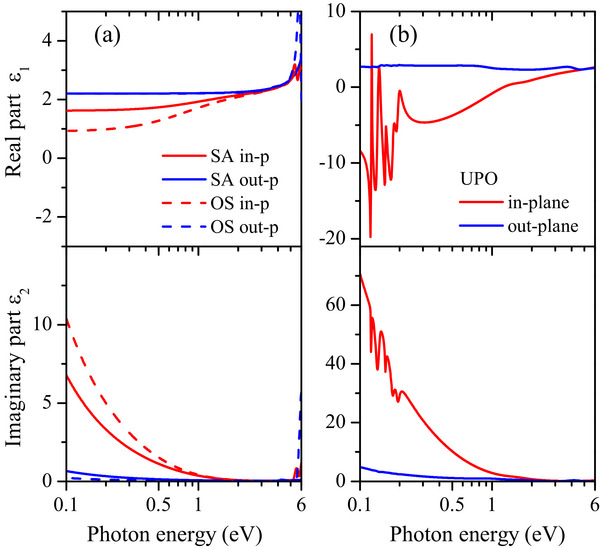
Complex dielectric function for SA and OS (a, left panels) and UPO series (b, right panels) thin films: in‐plane and out‐of‐plane components of their real (*ε_1_*) and imaginary (*ε_2_*) parts. Please note the different scale values used in panels (b).

The spectroscopic response of conjugated polymers in the visible spectral range is dominated by π–π* transitions due to delocalized electrons. However, several additional transitions can be observed at higher energies as due to both localized and delocalized states, as well as to chain terminals [65, 67, 68, 69, 70]. Therefore, extension of the spectral range to the UV spectral region is desirable. On the other hand, the low energy region of near‐ and mid‐infrared becomes essential to analyse the quasi‐free carrier dielectric response, playing a major role in conducting PEDOT. To this end, for all three series—SA, OS, UPO PEDOT samples—we extended the SE data by near‐normal reflectance/transmittance spectra up to 6.5 eV, and with FTIR polarized reflectance and transmittance spectra (0.1–1 eV) at different angles of incidence from near‐normal to 80 degrees (see Experimental section/Methods section for details). In Figure S5 we report the typical SE, R, and T spectra for the UPO thin film, 108 nm in thickness, which are simultaneously best fit from the dielectric modelling through the WVASE software. The overall dielectric functions for all the sample series are summarized in Figure 3.

We underline that for each material series, we investigated a high number of sample repetitions, this statistic giving us the possibility to validate the optical model adopted. In fact, in Table S1 we report the results of UPO multi‐sample analysis by increasing the spin coating deposition velocities, then assessing different film thickness values.

Moreover, in Figure S6 we report the overall ε_1_ and ε_2_ spectra for all sample series—with their in‐plane and out‐of‐plane components—as compared to their Drude‐like contribution only (from Equation 2 with $\varepsilon_{\infty }$= 0). In fact, an additional interesting parameter derivable from ε_2_ is the sum rule for solids Σ in Equation 4 [57], i.e. a measure of the e.m. energy absorption in the material at all the investigated frequencies.

(4) $$\Sigma =\int_{\omega_{\min}}^{\omega_{\max}}\omega \varepsilon_{2}\left(\omega \right)d\omega$$

We evaluated this quantity for both the dielectric response component—in‐plane and out‐of‐plane—with two modalities: integrating over all the imaginary part ε_2_ of the dielectric function (resulting value is named Σ^TOT^) or on the ε_2_ Drude‐like contribution only (hereafter named Σ^D^).

Then, in Table 2 we report a summary of significant parameter values of the sample series, e.g. the static optical conductivity, its anisotropy, the 4PP sheet conductivity, the in‐plane and out‐of‐plane Drude parameters, and the Σ^D^ / Σ^tot^ ratio. Similar data from the literature references are also reported.

As for the SA and OS series (Figure 3, panels a), we note that the MIR extended data clearly confirm the birefringent behaviour, as previously observed in the SE data only. The Drude‐like oscillator contribution inserted in the best‐fit modelling of the overall data allows us to more accurately assess the plasma frequency ω_p_ and the relative energy broadening Γ, correlated to quasi‐free carrier scattering mechanisms. As for the UPO PEDOT, the UV–vis dielectric response is similar to the commercial series, while the electronic conduction contribution is huge and dominates the in‐plane dielectric response (Figure 3, panel (b), note the increased scale values). A clear in‐plane vs out‐of‐plane conduction anisotropy is observed, the UPO PEDOT is about 10 times more conductive than commercial PEDOT:PSS formulations. From the Σ^D^/ Σ^tot^ ratio, the Drude contribution to the in‐plane response is about 90% for the UPO PEDOT, while lower values (even though meaningful) are observed for commercial PEDOT:PSS thin films. For the out‐of‐plane components, lower and scattered Drude contributions are verified.

For the different sets of UPO samples introduced in Table 1, the in‐plane and out‐of‐plane static optical conductivity values, estimated from the best‐fit, are reported in Table S1. We observe that despite their different thicknesses, samples S2–S4 exhibit very similar conductivity values, whereas sample S1 shows significantly lower one. This difference is attributed to the presence of water in the oxidising solution. Specifically, to achieve a higher Vanadium concentration for this sample, the oxidizing solution was prepared using a mixture of MSA and concentrated H_2_SO_4_ as the solvent. Since sulphuric acid contains residual water, traces of it remain in the final solution. In oxidative chemical polymerization, the amount of water present during polymerization is a critical parameter. Its presence is essential during the reaction because it acts as a proton scavenger. However, excessive quantities might have a detrimental effect on the film's characteristics [71]. In fact, a relevant water content has been correlated with increased efficiency in the initiation of the polymerization, while resulting in the formation of a higher number of polymer chains with lower molecular weights and, consequently, lower crystallinity [72].

We note the 4PP‐derived sheet conductivity is typically higher in value than the optical in‐plane one. This often happens in the comparison between the two different quantities, as it is due to the physical effects involved, the dipole coupling in *e.m*. radiation‐ film interaction and the four‐point contact to the thin film, respectively. Nonetheless, we confirm that the increasing trend from S1 to S3, and to S2–S4 is confirmed in the two experiments.

In addition, we tentatively adopt thermal and solvent annealing post‐growth of SA thin films and observe that pristine conductivity should be enhanced by a factor of 3 with thermal annealing at 100°C for 1 h duration and a factor of 100 by DMSO solvent annealing [73]. We underline that with the UPO PEDOT we were able to reach conductivities of the order of 30 kS/m, with no need of post‐growth processing. In Table 2, similar data from the literature are also reported. This result should be assigned to the remarkable difference between commercial PEDOT:PSS samples and UPO PEDOTs. In commercial PEDOT:PSS suspensions, short PEDOT chains are attached to insulating PSS chains via coulombic interaction. This causes the spontaneous formation of a morphology comprised of conductive PEDOT‐rich cores surrounded by PSS‐rich shells, due to their difference in water solubility. The PSS shell—if not removed by post‐deposition treatments—limits film conductivity. The absence of insulating PSS in the UPO PEDOT samples and the use of a mesylated counterion with a low molecular weight, which allows for the formation of a more crystalline structure, promoting intermolecular interactions, and therefore increasing the macroscopic conductivity and anisotropy of the electronic properties.

Finally, a specific comment is deserved for the medium infrared spectral region where vibrational resonances are overlapped to the quasi‐free carrier contribution, and a clear difference between commercial formulations and UPO PEDOT is observed.

For SA and OS series, the MIR response is ascribable to free‐carrier contribution only, as the vibrational contribution below 3000 cm^−1^ is from the quartz glass substrate (Figure S7a,b). We conclude that the vibrational contribution of thin films is negligible to the overall optical response. For UPO series the vibrational fingerprints are clearly observed for both in‐plane and out‐of‐plane dielectric functions, which deserve a future specific investigation. Here, we preliminarily note that the vibrational signal in UPO samples is much more intense with respect to PEDOT:PSS samples, even though thickness values are comparable. This suggests an enhancement of the IR signal occurs, presumably due to interaction with quasi‐free charges similar to what appears in Surface Enhanced InfraRed Spectra [74]. Moreover, notice that the IR spectrum of UPO samples (Figure S7c) seems to be characterized by Fano‐like anti‐resonances, which deeply modify the typical dispersive lineshape associated with the vibrational modes. In conjugated polymers the same effect appears when localized excited states are spectrally overlapped with broad polaronic bands [75]. In the present case, the high doping‐induced charge density of UPO series modifies the MIR spectrum of the undoped system generating intense IRAV modes (infrared activated vibrations) overlapped to the broad free‐carrier absorption background [74].

### 2.2 GIWAXS Analysis on PEDOT Films

**FIGURE 4:**
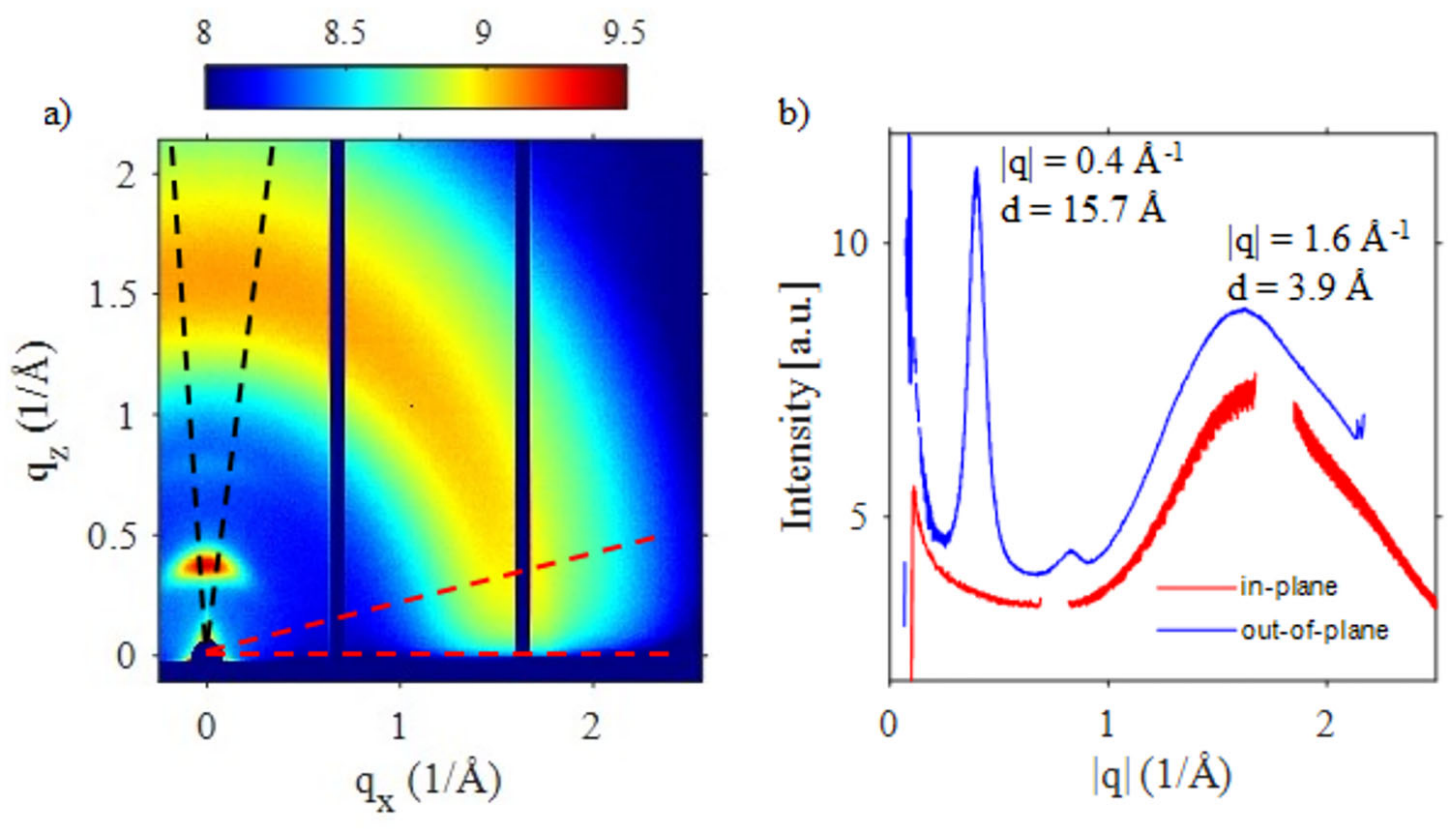
(a) False color GIWAXS map of a PEDOT UPO S4 sample. (b) In‐plane and out‐of‐plane profiles extracted by integrating the signal over the angle comprised between the red dashed line (in‐plane) and black dashed line (out‐of‐plane), respectively. The relevant peaks are labeled with the position in reciprocal |q| coordinate and the corresponding real space interplanar distances.

To get additional insights into the molecular arrangement of our PEDOT films, and to accomplish the uniaxial optical modelling adopted in the SE analyses, we performed GIWAXS experiments (details in the Experimental section) on a typical UPO S4 sample. The GIWAXS map (Figure 4a) and the in‐plane and out‐of‐plane profiles extracted (Figure 4b) show an isotropic broad peak centered at about q = 1.6 Å^−1^, together with a narrower peak in the out‐of‐plane direction centered at q = 0.4 Å^−1^, followed by a weak satellite at about 0.8 Å^−1^. The broad peak can be assigned to π–π stacking, with an average molecular distance of 3.9 Å. Its broadening is indicative of some disorder occurring in the stacking. The narrow and intense peak at 0.4 Å^−1^ is assigned to a family of reflections corresponding to an ordered lamellar structure perpendicular to both the PEDOT backbone and the PEDOT plane direction (out‐of‐plane with respect to the substrate) with periodicity d = 15.7 Å. Moreover, the weak satellite peak, due to its position and intensity, is assigned to the second order of the same plane reflections. All these findings are in full agreement with previous reports, which are summarized in Table 3 [76, 77, 78, 79, 80, 81, 82, 83].

**TABLE 3:** GIWAXS parameter values obtained on a typical UPO PEDOT sample and from the literature references.

Sample/Ref.	Interlamellar structure		π stacking structure	
	q (Å^−1^)	d(Å)	q (Å^−1^)	d(Å)
UPO PEDOT	0.4	15.7	1.6	3.9
Ref. [81]	0.4	15.7		
Ref. [82]	0.44	14.3		
Ref. [77]			1.55–1.82	3.45–4.05
Ref. [83]	0.45	14	1.77	3.54
Ref. [79]	0.44	14.3	1.86	3.4
Ref. [80]		13.9		3.4

**FIGURE 5:**
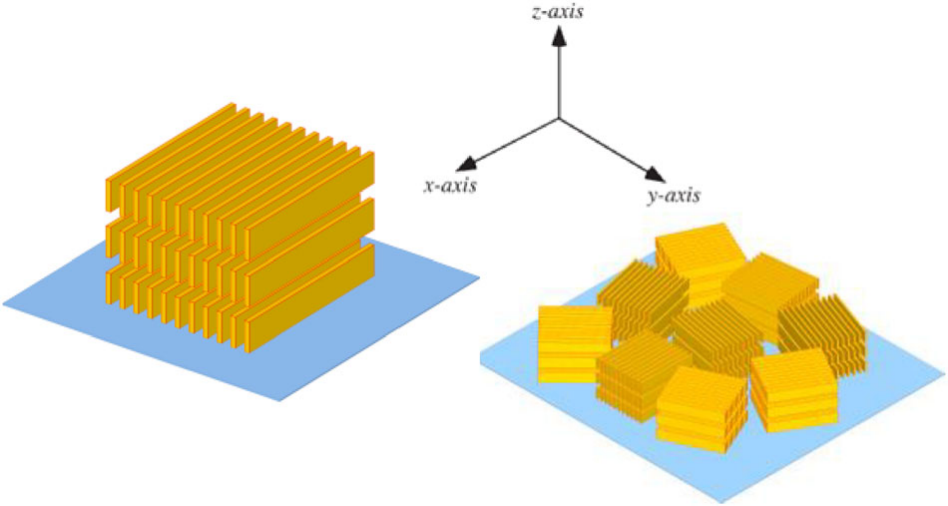
Sketch of the PEDOT lamellar crystallite (left) and its random assembly (right). Polarizability and charge transport in‐plane are favored over the corresponding out‐of‐plane response.

The GIWAXS characterization suggests that a second‐level anisotropy should be considered, reflecting the PEDOT material microstructure. Semicrystalline rigid polymers often exhibit rigid‐rod‐like orientation behavior; in our case they are likely to adopt a stacked lamellar structure in ordered regions, which is tentatively sketched in Figure 5. However, from our data we cannot exclude vertical inhomogeneities or stratifications occurring during growth, as evidenced from similar literature references [78, 79, 80].

In the ellipsometric‐based modelling, a more complex anisotropic response should be reproduced by a biaxial dielectric function, depending on the set of Euler angles (*θ, ψ, ϕ*) that allows for the precise location of the thin film optical axis of the thin films. Here, we preliminarily tried this approach by adopting the following configuration:

- for the azimuthal position (i.e. *ϕ*) of the sample during data acquisition, we align the sample with the plane of incidence on a radial direction of spin‐coating deposition;
- for the surface orientation of the PEDOT microstructure, we consider the polar angle *θ *and the azimuth* ψ *values as free‐variable parameters of the best fit to experimental UPO spectra. We remind that the polarized nature of SE, coupled to the extended interval of angles of incidence (from 20° to 75°), allows for observing the microstructure inclination with enhanced accuracy.

The dielectric function best‐fit results for the biaxial film modelling of UPO films point to a very small value of *θ* (typically < 1 deg) and *ψ* values of about 45 deg. We underline that for very low *θ* values the azimuth angle ϕ and ψ rotation become equivalent. Moreover, the dielectric function components in the backbone chain (x direction in our frame of reference) and along π stacking (our y direction) are very similar in values (see Figure S8, where vibrational contribution is omitted), equally contributing to the conduction process, providing an overall Drude weight of about 93% of the total response; the out‐of‐plane Σ component is instead composed of ∼40% Drude and ∼60% dielectric (see Table 2).

The biaxial tentative analysis is in good agreement with GIWAXS results, and the observed anisotropy can be interpreted by a preferential molecular orientation of the lamella lying almost parallel to the normal direction of the substrate plane.

## 3 Conclusions

The multidisciplinary investigation of the optical properties of PEDOT thin films polymerized in the liquid phase during spin‐coating reveals a pronounced uniaxial anisotropy of the complex refractive index, in full agreement with grazing‐incidence wide‐angle X‐ray scattering data and electrical resistivity measurements. A similar but lower optical anisotropy is also observed in commercial PEDOT:PSS formulations, indicating both this behavior as a general feature of PEDOT‐based conducting polymers prepared via oxidative chemical polymerization, as well as the power of ellipsometry to analyze in detail the optical response of complex uniaxial materials. Results here reported constitute a fundamental preliminary step toward engineering photonic crystal structures and metamaterials patterning PEDOT films via nanosphere lithography. Indeed, the performance of such nanostructures critically depends on the optical path of light within the material, which in turn requires detailed and reliable knowledge of the optical functions, particularly in the presence of anisotropy. As optical data for PEDOT, and more generally for anisotropic conjugated polymer thin films, are rarely reported and are strongly dependent on film preparation and processing conditions, our holistic work combining an extended spectroscopic analysis with electrical resistivity and structural characterization represents a significant advance toward a deeper understanding of the electronic and photonic properties of this distinctive class of macromolecules.

## 4 Experimental Section/Methods

### 4.1 Materials

3,4‐ethylenedioxythiophene (EDOT), vanadium pentoxide (V_2_O_5_), pyridine (Py), methanesulfonic acid (MSA), acetonitrile (MeCN), sulphuric acid (H_2_SO_4_, 96 wt.%) and hydrogenperoxide (H_2_O_2_, 30 wt.%) were purchased from Sigma‐Aldrich. All reagents were used as received without purification.

### 4.2 Thin Film Preparation

Three series of thin films were obtained on different reference substrates (QX quartz glass by Hellma, glass substrates, obtained by cutting 15 × 15 mm pieces from Corning microscope slides, and Silicon slides about 2×2 cm). Before deposition, the substrates were cleaned and conditioned by immersion in a piranha solution at 80°C for 40 min, then rinsed thoroughly with Milli‐Q water and dried with nitrogen flow at room temperature.

First, commercial PEDOT:PSS aqueous dispersion from different companies has been used as received to prepare thin films: in particular, Ossila Ltd HTL Solar (CAS Number 155090‐83‐8) with concentration 1.0–1.3 wt.% and Sigma‐Aldrich product number 655201 sold as “high‐conductivity grade” with concentration 3.0–4.0 wt%. PEDOT:PSS solutions were first filtered through a 0.45 µm PTFE syringe filter and then spin‐coated at 60, 80, or 100 rps for 60 s on glass substrates to tune the thickness. The films were then annealed on a hot plate at room atmosphere at 120°C for 20 min and then were immersed in methanol for 10 min. Then, films were dried at 120°C for 5 min. This treatment partially removes the insulating PSS component, increasing the ratio of conductive PEDOT and facilitating the formation of a more compact layer.

A sequential solution polymerization method [51] was used to synthesize UPO PEDOT thin films, with Vanadium pentoxide (V_2_O_5_) serving as the oxidant. In details, three oxidant precursor solutions were prepared, with different concentrations of V_2_O_5_. In particular, 0.2 and 0.3 M solutions were obtained by adding 0.45 and 0.65 g of V_2_O_5_ respectively to 12 mL of MSA. 0.4 M solution was obtained by adding 0.87 g of V_2_O_5_ to 12 mL of a 3:1 (by volume) mixture of MSA and H_2_SO_4_. Adding H_2_SO_4_ to the solvent mixture results in the presence of approximately 1.2 wt.% water in the oxidizing solution. The obtained suspensions were placed in vials, shaken for 24 h, and then stored for three months to allow for the complete solubilization of the V_2_O_5_. Since solubilization of V_2_O_5_ is an exothermic process, the vials were stored at constant room temperature because an increase in temperature would hinder the process. [53, 84] Prior to use, the concentration of V_2_O_5_ was determined by potentiometric titration using a standard iron (II) solution, in accordance with the procedure described elsewhere. [75] The oxidant solutions were then prepared by adding 420 µL of pyridine to 900 µL of the appropriate oxidant precursor solution. The monomer solution was prepared by diluting EDOT in acetonitrile at 30% v/v.

The PEDOT films were prepared as follows: the oxidant solution was deposited onto the treated substrates by spin‐coating at 3000 rpm for different times (Table 1). Then, without stopping the rotation, 200 µl of monomer solution was spin‐cast for a further 4 s on the same substrate.

Immediately after adding the monomer, the oxidant solution's yellow color rapidly turned dark blue, indicating polymerization of EDOT and formation of PEDOT films. Under these conditions, the polymerization process occurred almost instantaneously. After the films had formed, the samples were washed with methanol to remove any residual monomer and then annealed at 80°C for 10 min to improve film stability.

Optical profilometry images were obtained using Sensofar S Neox optical profiler with a confocal microscope equipped with a 20x objective, NA = 0.4. AFM microscopy images were obtained by Cypher ES microscope by Oxford Instruments, operating in tapping mode.

### 4.3 Optical Spectroscopy and Ellipsometry

Spectroscopic ellipsometry measurements were performed using a VASE instrument (J. A. Woollam Co., Lincoln, NE, USA) in the range 0.5–5 eV (in step of 10 meV) at different angles of incidence from 20 to 75° on thin films deposited on different substrates. A focusing probe adapter was used, providing a 200–300 microns diameter wide spot (depending on the incidence angle). A double‐beam Varian Cary 6000i dispersive spectrophotometer in the spectral range of 190–1800 nm (in step of 1 nm) was used to measure both reflectance and transmittance at near‐normal incidence. Quartz glass, amorphous glass, and Silicon reference substrates (about 20×20 mm size) were characterized before sample investigations.

Adopting a simultaneous best‐fit procedure to the SE, R and T spectra, the thin film complex refractive index dispersion was evaluated using the WVASE32 software (J. A. Woollam Co.) and adopting physical oscillator models that guarantee Kramers−Kronig consistency (see text for detailed dielectric modelling).

### 4.4 Infrared Spectroscopy and Surface Conductivity

IR *s*‐ and *p*‐polarized reflectance spectra were recorded with a step scan Bruker FTIR 66S Fourier transform infrared spectrometer equipped with HgCdTe detector to cover a broad spectral range (500–6000 cm^−1^). All measurements were performed at room temperature and at different angles of incidence. Sheet resistance measurements were performed by Ossila four‐point probe system with probe spacing 1.27 mm. System calibration is performed on the reference 100 nm ITO‐coated glass substrate (20×15 mm in size). From IR spectra, no clear evidence of residual catalyst has been found.

### 4.5 GIWAXS Maps

The measurements have been performed under the following experimental conditions: integration time 2 s, photon energy 12 keV corresponding to a wavelength of 1.033 Å, beam size 20 µm, incident angle 0.4°, with the detector placed at 4208 mm far from the sample.

## Funding

This research was funded by the Italian Ministry of University and Research (MUR) through the PRIN2020 project Polymer mETamateriALs for nanophotonicS – PETALS – code 2020TS9LXS.

## Conflicts of Interest

The author declares no conflicts of interest.

## Supporting information




**Supporting File**: marc70271‐sup‐0001‐SuppMat.docx.

## Data Availability

The data that support the findings of this study are available from the corresponding author upon reasonable request.
