# Supplementary material for: Spectroscopic Ellipsometry of Conducting Anisotropic Pedot thin Films
Source: Macromol Rapid Commun. 2026 Mar 30;47(11):e00965. doi: 10.1002/marc.202500965 (PMC13238304; doi:10.1002/marc.202500965)

marc.202500552- Supporting Information

SPECTROSCOPIC ELLIPSOMETRY OF CONDUCTING ANISOTROPIC PEDOT THIN FILMS

Francesco Bisio, Katia Sparnacci
Dipartimento di Scienze e Innovazione Tecnologica, Università degli Studi del Piemonte Orientale, Alessandria, Italy

Angelo Angelini

Advanced Materials Metrology and Life Science Division, INRiM Institute (Istituto Nazionale di Ricerca Metrologica), Torino, Italy

Martina Martusciello, Daniela Di Fonzo, Davide Comoretto

Dipartimento di Chimica e Chimica Industriale, Università degli Studi di Genova, Genova, Italy

Maddalena Patrini *

Dipartimento di Fisica Alessandro Volta, Università degli Studi di Pavia, Pavia, Italy

E-mail: maddalena.patrini@unipv.it

**Figure S1**. Optical profilometry analysis: optical images for samples UPO S1-S4.


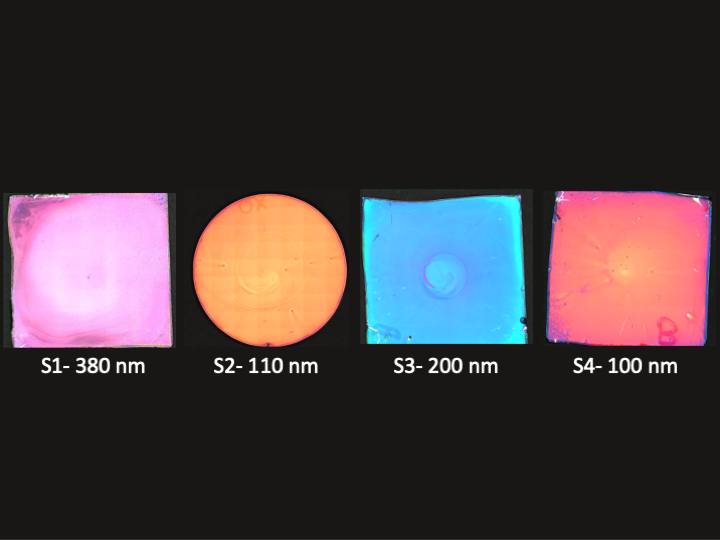


**Figure S2**. Optical profilometry analysis example for UPO S2 sample: a) interferometric image acquired with a20x microscope objective (NA = 0.4); b) surface region extracted from the blue square in a) that shows a zoom on the surface roughness; c) surface profile extracted along the blue dashed line in b).


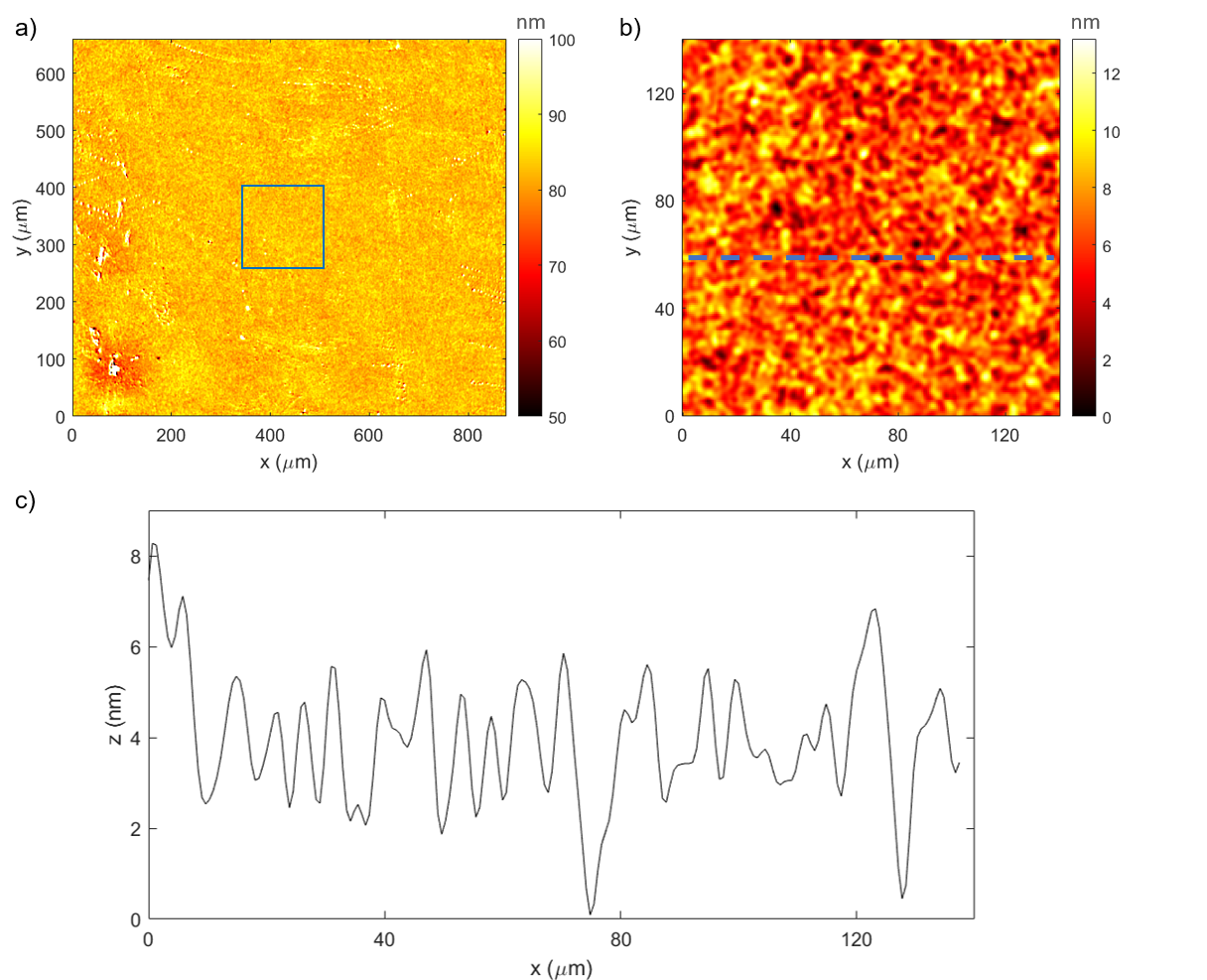


**Figure S3.** Atomic force microscopic height (A) and phase (B) images obtained for UPO S2 sample in 1 μm x 1μm area. The determined surface roughness rms value is 1.7 nm.


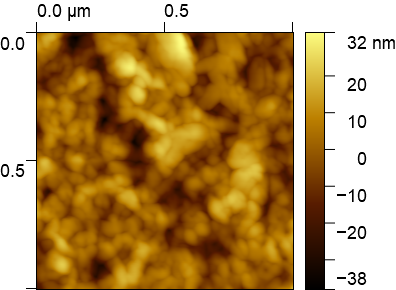

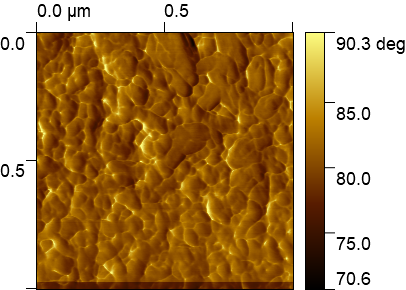


B

A

**Table S1.** Thin film growth and structural parameter values for the three series investigated.

| Sample  series | Spin coating deposition velocity [rps] | Optical  Profilometry thickness (nm) | SE Film thickness  [nm] | % difference between SE and profilometry | In-plane optical conductivity [10^3^ S/m] | Out-of-plane optical  conductivity  [10^3^ S/m] | Conductivity anisotropy | Sheet 4PP conductivity  [10^3^ S/m] |
| --- | --- | --- | --- | --- | --- | --- | --- | --- |
| SA | 60 |  | 141 |  | 1.55 | 0.147 |  | 0.3 |
|  | 80 |  | 121 |  | / | / |  | / |
|  | 100 |  | 89 |  | / | / |  | / |
| OS | 60 |  | 162 |  | 2.53 | 0.058 |  | 0.2 |
|  | 80 |  | 142 |  | / | / |  | / |
|  | 100 |  | 127 |  | / | / |  | / |
| UPO S1 | 50 | 380 | 377 | -0,8% | 8.39 | 4.31 | 2 | 1.6 |
| UPO S2 | 50 | 110 | 118 | 6.8% | 15.4 | 0.66 | 23 | 34.6 |
| UPO S3 | 50 | 200 | 236 | 15% | 13.5 | 1.92 | 7 | 32.3 |
| UPO S4 | 50 | 100 | 108 | 7.4% | 16.0 | 1.28 | 12 | 33.7 |

**Figure S4.** In-plane and out-of-plane components of the complex refractive index - real (*n*, upper panels) and imaginary (*k*, lower panels) part - for SA(a), OS(b) and UPO (c) thin films.

**Figure S5**. Typical SE spectra (Ψ and Δ, $\frac{r_{p}}{r_{s}}=\tan\left( \Psi\right)e^{i\Delta}$, where r_p_ and r_s_ are the complex reflection Fresnel coefficients for p and s polarization, respectively, at different angles of incidence, near-normal reflectance (5°) and normal incidence transmittance spectra, as measured on UPO S4 sample.

**
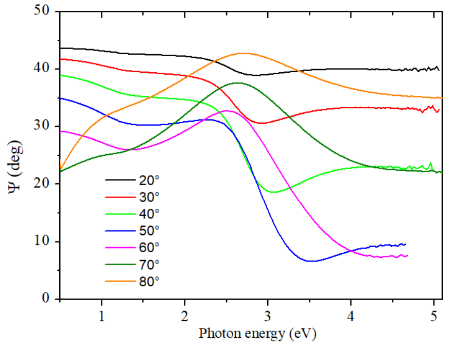

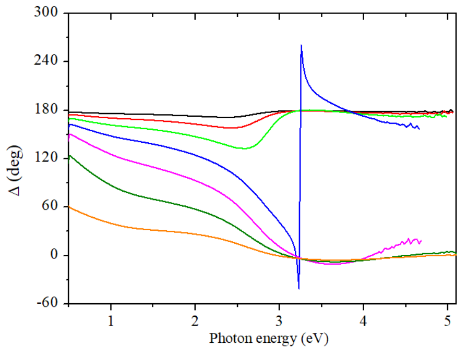
**

**
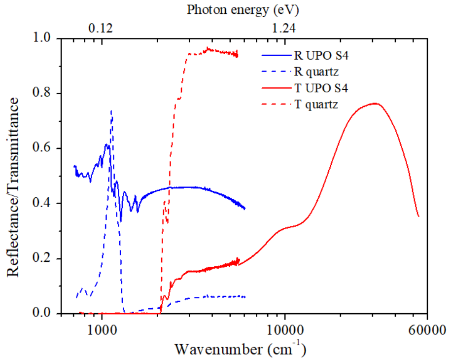
**

**Figure S6.** In-plane and out-of-plane complex dielectric function spectra for the different sets of UPO series thin films (see text). The overall functions are reported in solid lines, while the Drude contribution only is in dashed lines (see text).


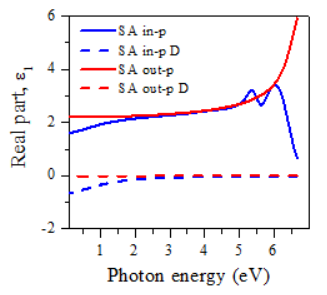

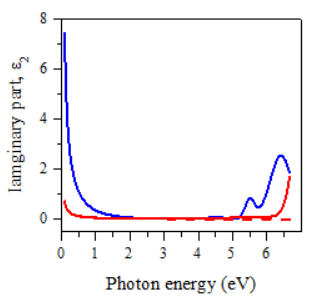


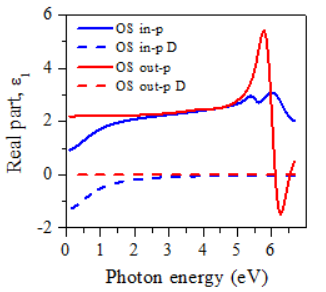

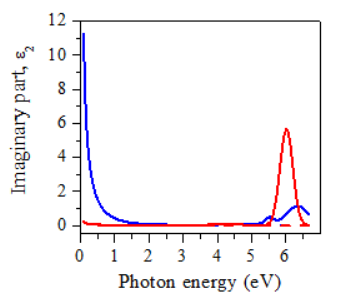


**
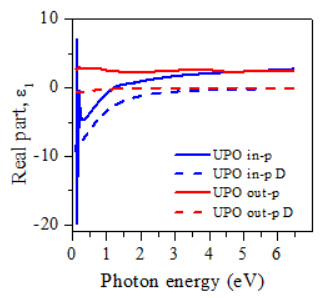

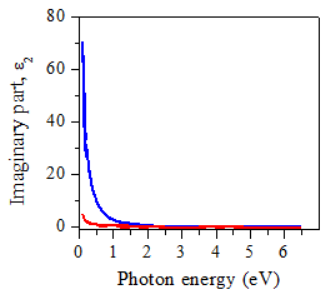
**

**Figure S7.** Medium Infrared Polarized Reflectance Spectra of PEDOT samples investigated in this work: a) SA series; b) OS series; c) UPO PEDOT series

**Figure S8.** Biaxial model dielectric functions for the UPO series-S4 set sample (see text).


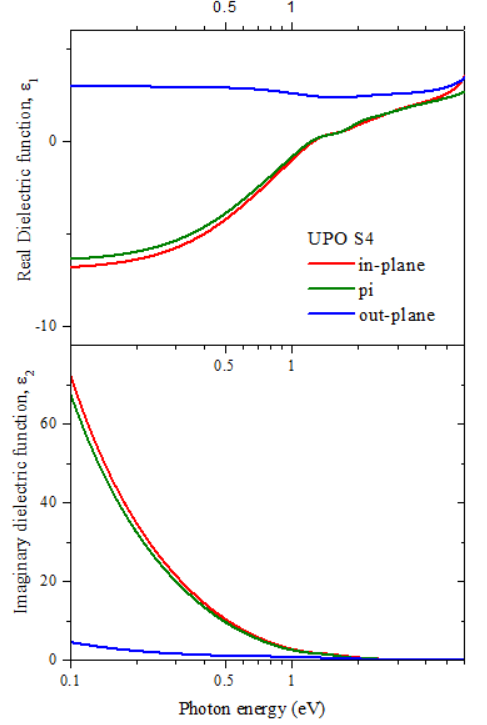

Supplement: Supplementary file 1 — Supporting File: marc70271‐sup‐0001‐SuppMat.docx. [file MARC-47-e00965-s001.docx]
